# Early-pregnancy N-terminal pro-brain natriuretic peptide level is inversely associated with hypertensive disorders of pregnancy diagnosed after 35 weeks of gestation

**DOI:** 10.1038/s41598-024-63206-5

**Published:** 2024-05-28

**Authors:** Masaya Takahashi, Luka Suzuki, Nanase Takahashi, Mayu Hanaue, Masahiro Soda, Tamito Miki, Naoko Tateyama, Shiro Ishihara, Taro Koshiishi

**Affiliations:** 1 Hagukumi Maternal and Child Clinic, Kanagawa, Japan; 2 Department of Medicine, Diabetes Center, Quantitative Biosciences Institute (QBI), UCSF (University of California San Francisco), San Francisco, CA USA; 3 Department of Metabolism and Endocrinology, Juntendo University Graduate School of Medicine, Tokyo, Japan; 4 Department of Cardiology, Saitama Medical Center, Saitama Medical University, Saitama, Japan

**Keywords:** Hypertensive disorders of pregnancy, Preeclampsia, N-terminal pro-brain natriuretic peptide levels, Predictive marker, Predictive value, Predictive markers, Pregnancy outcome

## Abstract

Hypertensive disorders of pregnancy (HDP) are among the major causes of high maternal and fetal/neonatal morbidity and mortality rates. Patients with HDP have significantly elevated N-terminal pro-brain natriuretic peptide (NT-proBNP) levels at diagnosis; however, the NT-proBNP levels during early pregnancy are largely unknown. This study aimed to validate the association between HDP and NT-proBNP levels. This retrospective study evaluated 103 pregnant women who developed HDP diagnosed after 35 weeks of gestation and 667 who did not. The HDP group had significantly lower early-pregnancy NT-proBNP levels than the without HDP group. However, the two groups did not significantly differ in terms of the late-pregnancy NT-proBNP levels. After adjusting for confounding factors such as age, body mass index, parity, and blood pressure levels, high early-pregnancy NT-proBNP levels were associated with a lower HDP risk. Early-pregnancy NT-proBNP levels ≥ 60.5 pg/mL had a negative predictive value of 97.0% for ruling out HDP, with a sensitivity of 87.4% and specificity of 62.5%. In conclusion, elevated early-pregnancy NT-proBNP levels were associated with a lower HDP risk. Moreover, a cutoff point of ≥ 60.5 pg/mL for early-pregnancy NT-proBNP levels had a high negative predictive value and sensitivity for ruling out HDP. These findings can provide new clinical implications.

## Introduction

Hypertensive disorders of pregnancy (HDP) are associated with high maternal and fetal/neonatal morbidity and mortality worldwide, with an incidence rate of 5%–25%^1–4^. Hence, they are significant concerns in the field of obstetrics. The exact etiology of HDP remains unknown. However, several factors play important roles^5,6^. To prevent or delay the onset of preeclampsia, low-dose aspirin is commonly recommended during pregnancy by several medical societies, including the American College of Obstetricians and Gynecologists^7^. The American College of Obstetricians and Gynecologists issued the Hypertension in Pregnancy Task Force Report, which recommends daily low-dose aspirin administered starting at least in the late first trimester for women with a history of early-onset preeclampsia and preterm delivery at < 34 0/7 weeks of gestation, or for women with more than one prior pregnancy complicated with preeclampsia^7^. However, in the absence of high-risk factors for preeclampsia, current evidence does not support the prophylactic use of low-dose aspirin for preventing early pregnancy loss, fetal growth restriction, stillbirth, or preterm birth. Thus, among low-risk or nulliparous women, a practical and effective treatment for HDP has not been identified. In fact, a delayed decision to deliver is often associated with high fetal/neonatal morbidity and mortality rates^1,2,4^.

Early prediction and diagnosis of HDP are essential for timely intervention and better maternal and fetal outcomes. In relation to this, circulating biomarkers have gained increasing attention. Previous studies have shown that N-terminal pro-brain natriuretic peptide (NT-proBNP) and BNP levels can be possible predictors of HDP^8–12^. NT-proBNP and BNP have been considered as diagnostic markers of subclinical cardiac dysfunction^13^. The byproduct of BNP is biologically inactive NT-proBNP, which has a greater sensitivity for detecting left ventricular dysfunction^14^. Approximately 25% of BNP is excreted unmodified by the kidneys. The remaining BNP is eliminated after binding to the receptor or via enzymatic degradation. Conversely, NT-proBNP can only be excreted passively mainly by the kidney. Due to their various clearances, NT-proBNP has a longer half-life (120 vs. 20 min) and a higher plasma concentration (approximately 6 times) than BNP^15^, which indicates the stability of NT-proBNP. Additionally, the concentration range of NT-proBNP has a broader clinical span compared with that of BNP (0–35,000 vs. 0–5000 pg/mL)^16^. Therefore, NT-proBNP can more accurately reflect clinical conditions than BNP. Previous studies have focused on NT-proBNP levels during HDP diagnosis^17–20^. On the other hand, few studies have shown NT-proBNP levels prior to the development of HDP. The Nulliparous Pregnancy Outcomes Study: Monitoring Mothers-to-Be (nuMOM2B) showed that low first-trimester NT-proBNP levels were associated with a high risk of developing HDP^21^. However, research examining NT-proBNP levels during early pregnancy in the context of multiparous women remains scarce.

Considering the potential implications of early prediction and intervention in HDP, we conducted a retrospective study involving pregnant women, including, for the first time, multiparous women in our primary care hospital and evaluated the association between early-pregnancy NT-proBNP levels and subsequent HDP development. We compared the plasma NT-proBNP concentrations between patients who developed HDP and those who did not. Second, we used logistic regression models, adjusting for confounding factors associated with HDP to explore the association between early-pregnancy NT-proBNP levels and the onset of subsequent HDP diagnosed after 35 weeks of gestation. Finally, we aimed to elucidate the predictive value of this biomarker, providing valuable insights for clinical practice.

## Results

### Study population and clinical characteristics of the participants

**Figure 1:**
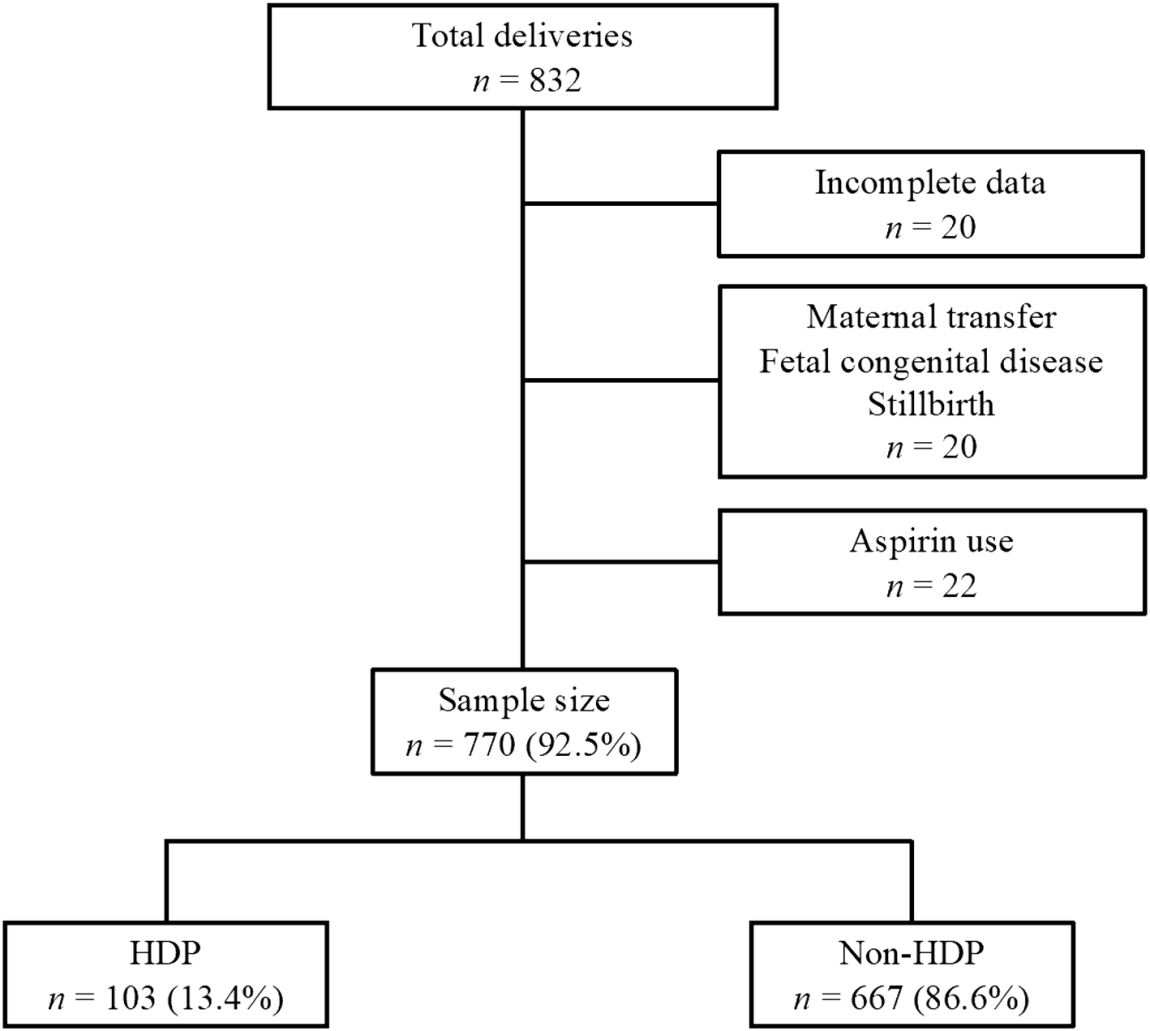
Flow diagram of the inclusion of study participants between April 2021 and March 2022. Patients were diagnosed with HDP based on the 2018 ISSHP criteria. HDP, hypertensive disorders of pregnancy.

This study included 832 women. Among them, 62 were excluded due to incomplete data sets (n = 20), maternal transfer (n = 16), fetal congenital disease (n = 1), stillbirth (n = 3), and aspirin use (n = 22). Thus, the remaining 770 (92.5%) patients were included in our analysis (Fig. 1). In total, 103 (13.4%) patients presented with HDP, and 11 patients were diagnosed with preeclampsia.

Table 1 shows the clinical characteristics of the study population. The HDP and without HDP groups were comparable in terms of maternal age, early- and late-pregnancy hemoglobin levels, gestational age at delivery, rate of painless delivery and cesarean delivery, birth weight, and umbilical artery pH. However, significant differences were observed in some parameters, such as early- and late-pregnancy body mass index (BMI) and systolic/diastolic blood pressure, parity, and blood loss volume.

**Table 1:** Pregnancy characteristics and maternal–fetal outcomes.

	Patients without HDP (n = 667)	Patients with HDP (n = 103)	*p* value
Maternal age (years)	33.1 ± 4.0	33.5 ± 4.3	0.30
BMI			
Early pregnancy	20.4 ± 2.3	20.9 ± 2.5	< 0.05
Late pregnancy	23.9 ± 2.4	24.5 ± 2.3	< 0.05
Parity			
Nullipara	294 (44.1%)	64 (62.1%)	< 0.001
Multipara	373 (55.9%)	39 (37.9%)	
Blood pressure (mmHg)			
Early pregnancy			
Systolic	109.0 ± 10.6	115.6 ± 11.0	< 0.0001
Diastolic	63.3 ± 8.1	69.2 ± 8.2	< 0.0001
Late pregnancy			
Systolic	110.6 ± 9.9	117.0 ± 11.1	< 0.0001
Diastolic	64.1 ± 7.9	69.6 ± 8.4	< 0.0001
Hb level (g/dL)			
Early pregnancy	12.8 ± 4.8	12.7 ± 1.0	0.29
Late pregnancy	11.0 ± 1.0	11.1 ± 1.1	0.38
Gestational age at delivery (weeks)	39.1 ± 1.0	39.4 ± 1.1	0.07
Painless delivery	514 (77.1%)	82 (79.6%)	0.56
Blood loss volume (g)	410 (65–3703)	490 (64–3100)	< 0.01
Cesarean delivery	58 (8.7%)	12 (11.7%)	0.33
Birth weight (g)	3026 (2104–4226)	3050 (1954–3940)	0.42
Umbilical artery pH	7.29 ± 0.1	7.29 ± 0.1	0.89

Data were presented as mean ± standard deviation, median with range, n (%).

*p* values < 0.05 were considered statistically significant.

*HDP* Hypertensive disorders of pregnancy, *BMI* Body mass index.

### Association between NT-proBNP levels and HDP

**Figure 2:**
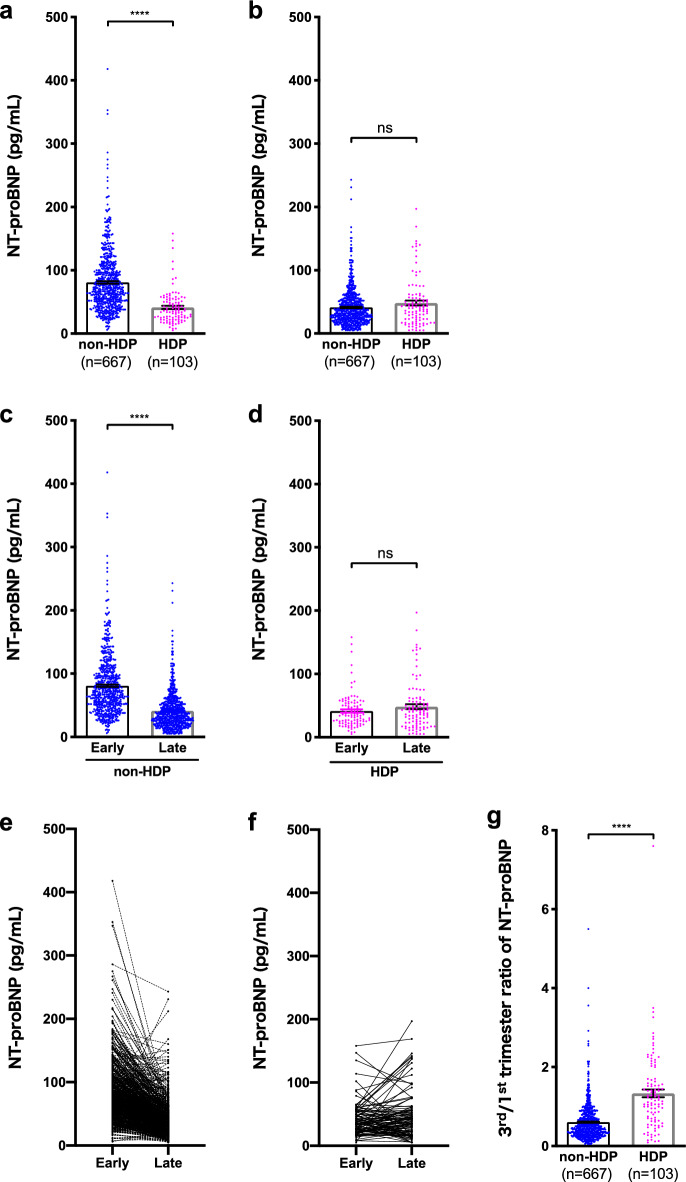
Comparison of the plasma NT-proBNP concentrations and the third-to-first ratio of NT-proBNP during the first and third trimester. (**a**) NT-proBNP levels during the first trimester (7–13 weeks of gestation). (**b**) NT-proBNP levels during the third trimester (35–37 weeks of gestation). (**c**) NT-proBNP levels in patients without HDP. (**d**) NT-proBNP levels in patients with HDP. (**e**) Transition of the NT-proBNP levels between early and late pregnancy in patients without HDP. (**f**) Transition of the NT-proBNP levels between early and late pregnancy in patients with HDP. (**g**) Ratio of third-to-first-trimester NT-proBNP levels. NT-proBNP, N-terminal pro-brain natriuretic peptide; HDP, hypertensive disorders of pregnancy. Data were presented as mean ± standard error of the mean. ****, *p* < 0.0001. ns, Not significant. Early pregnancy was defined as blood sampling during the first trimester (7–13 weeks of gestation). Late pregnancy was defined blood sampling during the third trimester (35–37 weeks of gestation).

In the early-pregnancy phase (7–13 weeks), the HDP group had significantly lower NT-proBNP levels than the without HDP group (41.4 [5–158] vs. 80.9 [6–418], *p* < 0.0001) (Fig. 2a and Table 2). However, during the late-pregnancy phase (35–37 weeks), no significant difference was observed in terms of the NT-proBNP levels between the two groups (48.2 [5–197] vs. 41.2 [5–243], *p* = 0.24) (Fig. 2b and Table 2). In patients without HDP, the NT-proBNP levels in the early-pregnancy phase were significantly higher than those in the late-pregnancy phase (80.9 [6–418] vs. 41.2 [5–243], *p* < 0.0001) (Fig. 2c). Meanwhile, in patients with HDP, the NT-proBNP levels were comparable between the two phases (41.4 [5–158] vs. 48.2 [5–197], *p* = 0.58) (Fig. 2d). The NT-proBNP levels of patients who did not develop HDP decreased in the late-pregnancy phase. Meanwhile, this decreasing trend was not evident in patients with HDP (Fig. 2e,f). In addition, the HDP group had a significantly higher ratio of third-to-first-trimester NT-proBNP levels than the without HDP group (1.53 [0.09–7.60] vs. 0.61 [0.05–5.50], *p* < 0.0001) (Fig. 2g and Table 2). Furthermore, 94 patients without HDP (14.1%) had early-pregnancy NT-proBNP levels > 125 pg/mL. However, only three patients with HDP (2.7%) reached this threshold. Among the patients exceeding the threshold, none presented with cardiovascular disease. Therefore, patients without HDP had elevated early-pregnancy NT-proBNP levels, which exceed the standard for nonpregnant adults. These levels then declined toward the late pregnancy. Using the logistic regression models and even after adjusting for confounding factors such as age, BMI, parity, and systolic/diastolic blood pressure levels, high early-pregnancy NT-proBNP levels were associated with a reduced risk of HDP (adjusted odds ratio [OR] 0.66; 95% confidence interval [CI] 0.60–0.74) (Table 3). This study excluded patients taking aspirin; however, some studies included these patients to account for confounding factors in HDP. Therefore, we confirmed the logistic regression models including the patients taking aspirin and analyzed them. This analysis, adjusted for confounding factors such as age, BMI, parity, systolic/diastolic blood pressure levels, and aspirin use, yielded similar results (adjusted OR 0.66; 95% CI 0.59–0.72) (Supplementary Table 1).

**Table 2:** Comparison of plasma NT-proBNP concentrations and the ratio of third-to-first-trimester NT-proBNP levels.

	Patients without HDP (n = 667)		Patients with HDP (n = 103)		*p* value
	Mean	Range	Mean	Range	
NT-proBNP concentrations (pg/mL)					
1st trimester (7–13 weeks of gestation)	80.9	6–418	41.4	5–158	< 0.0001
3rd trimester (35–37 weeks of gestation)	41.2	5–243	48.2	5–197	0.24
3rd/1st trimester ratio of NT-proBNP	0.61	0.05–5.50	1.53	0.09–7.60	< 0.0001

*p* values < 0.05 were considered statistically significant.

*NT-proBNP* N-terminal pro-brain natriuretic peptide, *HDP* Hypertensive disorders of pregnancy.

**Table 3:** Crude and adjusted association of the first-trimester NT-proBNP and HDP.

Measurement parameters	Crude OR (95% CI)	*p* value	Adjusted OR (95% CI)	*p* value
NT-proBNP levels (OR per 10 units)	0.66 (0.60–0.73)	< 0.0001	0.66 (0.60–0.74)^a^	< 0.0001

Data were presented as OR with 95% CI.

*p* values < 0.05 were considered statistically significant.

*NT-proBNP* N-terminal pro-brain natriuretic peptide, *HDP* Hypertensive disorders of pregnancy, *BP* Blood pressure, *OR* Odds ratio, *CI* Confidence interval.

^a^Adjusted for age, body mass index (in early pregnancy), parity, and systolic/diastolic blood pressure.

### Correlation analysis

The early- and late-pregnancy NT-proBNP levels were not correlated with maternal age, gestational age at delivery, blood loss volume, birth weight, and umbilical artery pH. However, a weak negative correlation was observed among early- and late-pregnancy BMI, early-pregnancy systolic/diastolic blood pressure, and early-pregnancy hemoglobin levels (Supplementary Table 2).

### Predictive value of NT-proBNP levels in HDP

**Figure 3:**
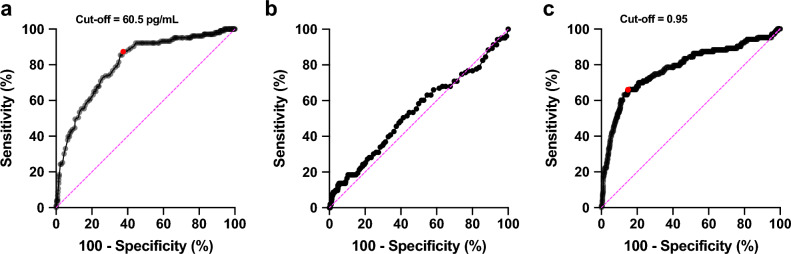
ROC curve for the accuracy analysis of NT-proBNP levels and the ratio of third-to-first-trimester NT-proBNP levels for predicting HDP. (**a**) NT-proBNP levels during the first trimester (7–13 weeks of gestation). (**b**) NT-proBNP levels during the third trimester (35–37 weeks of gestation). (**c**) Ratio of third-to-first-trimester NT-proBNP levels. ROC, receiver operating characteristic; NT-proBNP, N-terminal pro-brain natriuretic peptide; HDP, hypertensive disorders of pregnancy.

Figure 3, Tables 4 and 5 present the diagnostic performance of NT-proBNP levels for HDP. The optimal cutoff for early-pregnancy NT-proBNP levels was 60.5 pg/mL based on the receiver operating characteristic curve (area under the receiving curve [AUC] 0.806; Youden index: 0.499; *p* < 0.0001; 95% CI 0.762–0.851) (Fig. 3a and Table 4). This threshold yielded a sensitivity of 87.4% and a specificity of 62.5% (Table 4). The negative predictive value (NPV) for ruling out subsequent HDP using early-pregnancy NT-proBNP levels ≥ 60.5 pg/mL was 97.0% (95% CI 95.1–98.2). The negative likelihood ratio was 0.2 (95% CI 0.1–0.3) (Table 5).

**Table 4:** Diagnostic efficacy of the NT-proBNP levels and the third-to-first-trimester ratio of NT-proBNP for HDP.

	AUC (95% CI)	Sensitivity (%)	Specificity (%)	Youden index	*p* value
NT-proBNP levels					
1st trimester (7–13 weeks of gestation)	0.806 (0.762–0.851)	87.4	62.5	0.499	< 0.0001
3rd trimester (35–37 weeks of gestation)	0.536 (0.472–0.600)	50.5	58.6	0.091	0.24
3rd/1st trimester ratio of NT-proBNP	0.783 (0.728–0.839)	66.0	85.2	0.512	< 0.0001

*p* values < 0.05 were considered statistically significant.

*NT-proBNP* N-terminal pro-brain natriuretic peptide, *HDP* Hypertensive disorders of pregnancy, *AUC* Area under the curve, *CI* Confidence interval.

**Table 5:** Predictive value of the NT-proBNP levels and the third-to-first-trimester ratio of NT-proBNP for HDP.

	PPV (%)	NPV (%)	Positive LR	Negative LR
1st trimester NT-proBNP levels (7–13 weeks of gestation)	26.5 (24.2–29.0)	97.0 (95.1–98.2)	2.3 (2.1–2.6)	0.2 (0.1–0.3)
3rd/1st trimester ratio of NT-proBNP	40.8 (35.4–46.4)	94.2 (92.5–95.5)	4.5 (3.5–5.6)	0.4 (0.3–0.5)

Data were presented as values with 95% confidence interval.

*NT-proBNP* N-terminal pro-brain natriuretic peptide, *HDP* Hypertensive disorders of pregnancy, *PPV* Positive predictive value, *NPV* Negative predictive value, *LR* Likelihood ratio.

However, the late-pregnancy NT-proBNP levels did not have a superior predictive accuracy (AUC: 0.536; Youden index: 0.091; *p* = 0.24; 95% CI 0.472–0.600) (Fig. 3b and Table 4). The cutoff value for the ratio of third-to-first-trimester NT-proBNP levels was 0.95 (Fig. 3c), with an AUC of 0.783 (Youden index: 0.512; *p* < 0.0001; 95% CI 0.728–0.839), with a sensitivity of 66.0% and specificity of 85.2% (Table 4). The positive predictive value (PPV) for ruling in subsequent HDP using the ratio of third-to-first-trimester NT-proBNP levels ≥ 0.95 was 40.8% (95% CI 35.4–46.4), and the positive likelihood ratio was 4.5 (95% CI 3.5–5.6) (Table 5). The NPV for ruling out subsequent HDP using the ratio of third-to-first-trimester NT-proBNP levels < 0.95 were 94.2% (95% CI 92.5–95.5). The negative likelihood ratio was 0.4 (95% CI 0.3–0.5) (Table 5).

## Discussion

This retrospective study validates the association between NT-proBNP levels and HDP diagnosed after 35 weeks of gestation while exploring their potential clinical utility during pregnancy. Initially, logistic regression models revealed a significant inverse relationship between NT-proBNP levels and the incidence of HDP. Additionally, an early-pregnancy NT-proBNP level cutoff of ≥ 60.5 pg/mL demonstrated high NPV and sensitivity to exclude HDP. Moreover, a third-to-first-trimester NT-proBNP ratio of < 0.95 exhibited an observed NPV of 94.2% in predicting subsequent HDP.

First, patients with HDP had significantly lower early-pregnancy NT-proBNP levels compared with those without HDP. The logistic regression analyses showed that higher early-pregnancy NT-proBNP levels were associated with a lower HDP risk. There are numerous studies on elevated NT-proBNP levels immediately before and at HDP onset^20,22,23^. However, only a few studies focused on the early stages of pregnancy. To date, only one report that used logistic regression models has focused on the association between early-pregnancy NT-proBNP levels and HDP in nulliparous women. This study showed that higher early-pregnancy NT-proBNP concentrations were associated with a significantly lower risk of HDP and a lower risk of incident hypertension 2–7 years after delivery^21^. Hence, this is the first study that evaluated multiparous women with HDP who had low early-pregnancy NT-proBNP levels compared with those without HDP. The logistic regression models showed that higher early-pregnancy NT-proBNP levels were associated with a lower HDP risk. In several previous studies^7,21^, patients who took aspirin were included and analyzed as a confounding factor in HDP using multiple logistic regression. Consequently, we confirmed that the results remained similar even after their inclusion and analysis.

Incidentally, the detailed mechanism underlying the transition in NT-proBNP levels during pregnancy remains unclear. A recent study investigated the genetic associations between cardiovascular disease-related proteins and HDP using Mendelian randomization, which is a method used in genetic epidemiology. Their results showed that 10 proteins, including NT-proBNP, reflect pathways with potential roles in HDP development^24^. Considering that maternal conditions significantly occur in the early-pregnancy stages, our evaluation focused on early-pregnancy NT-proBNP levels. In fact, the average early-pregnancy NT-proBNP levels of women without HDP were approximately more than twice as high as that of those in nonpregnant Asian young women from a previous report (80.9 vs. 34.9 pg/mL)^25^. Indeed, based on a previous study, pregnant women in the first trimester had higher NT-proBNP levels than nonpregnant women in the United States^26^. This change occurred in women without HDP immediately after pregnancy. Therefore, a transient increase in early-pregnancy NT-proBNP levels can be important for maintaining hemodynamic homeostasis during pregnancy. In addition, our study showed that the early-pregnancy NT-proBNP levels of the HDP group was approximately half as low as those of the without HDP group. Lower NT-proBNP levels can indicate impaired early vascular adaptation to pregnancy, resulting in later-onset HDP. In normal pregnancies, decreased peripheral vascular resistance caused by elevated NT-proBNP levels and vascular sensitivity to the presser effects of angiotensin II and norepinephrine as compensatory mechanisms can facilitate adequate blood flow to the utero-placental circulation to ensure adequate intravascular volume during rapid hemodynamic changes that occur in the early stages of pregnancy. Therefore, a healthy maternal–fetal interface should be developed^27,28^. However, in patients with HDP who do not present with elevated NT-proBNP levels during early gestation, resulting in insufficient blood supply to the uteroplacental circulation, subsequent maternal hypertension can be a compensatory response to increased placental blood flow.

Further studies should be performed to discuss the detailed mechanisms underlying the source of NT-proBNP. High NT-proBNP levels are commonly associated with increased left ventricular end-diastolic dimension^29^. In contrast, the cardiac load increases toward the late term of pregnancy. The effect of this factor in early pregnancy is negligible. Thus, we focused on two possible factors, which are endogenous hormones related to pregnancy and organ development during pregnancy. In terms of endogenous hormones, hCG, which is released from the placenta and increases rapidly in early pregnancy, is one of the candidate hormones. Its peak is at approximately 10 weeks of gestation, and its production decreases toward late pregnancy. The beta subunit of hCG has structural homology with thyroid stimulating hormone; therefore, hCG can weakly stimulate its receptor, leading to the increased production of thyroidal hormone^30^. Hyperthyroidism increase BNP and NT-proBNP levels, and transient hyperthyroidism during early pregnancy is usually observed during prenatal checkups. Moreover, a previous study showed that the beta subunit of hCG in early pregnancy was significantly lower in pregnant women who subsequently develop preeclampsia^31^, which can be a reason for low early-pregnancy NT-proBNP levels in women with HDP. Taken together, early-pregnancy hCG levels can be a factor affecting NT-proBNP levels. The placenta is a unique organ that develops during pregnancy. Corin is an enzyme that converts BNP prohormone into BNP and NT-proBNP^32^. Previous rodent and human studies have revealed that NT-proBNP is secreted from the placenta, in addition to the ventricular muscle, to maintain pregnancy. In mouse placenta, BNP mRNA is expressed in the peripheral margin of the decidual layer^33^. In addition, corin mRNA was detected in the mouse uterus, umbilical cord, and placenta^34^. Moreover, corin-deficient mice developed high blood pressure and proteinuria late in gestation. However, their blood pressure and protein levels normalized after delivery^35^. In humans, the transcripts of corin and proBNP/NT-proBNP mRNA and their proteins have been observed in maternal spiral arteries and syncytiotrophoblasts in placental tissues^19,32,34,36–39^. Considering that the placenta is an endocrine organ that synthesizes and secretes hormones that maintain pregnancy status, NT-proBNP levels in maternal blood might be partly attributed to the placental release of the peptides.

Another pivotal finding was that a cutoff point of ≥ 60.5 pg/mL for early-pregnancy NT-proBNP levels had high NPV and sensitivity for ruling out HDP diagnosed after 35 weeks of gestation. If we focus on the predictive markers of HDP onset during the first trimester, there are conflicting findings regarding its usefulness. That is, some studies have shown that it is valuable. Meanwhile, other studies have claimed that it lacks predictive power because there are extremely few relevant studies^40–47^. Indeed, only one study including nulliparous women has been reported to date. In this study, NT-proBNP levels in the first trimester were significantly lower in patients with term preeclampsia^47^, which is consistent with our results. To the best of our knowledge, this is the first study that evaluated the predictive value of early-pregnancy NT-proBNP levels in both multiparous and nulliparous women. In this study, we suggested a cutoff point of ≥ 60.5 pg/mL for early-pregnancy NT-proBNP levels had a high NPV and sensitivity for ruling out HDP. Additionally, the observed NPV for ruling out subsequent HDP using a third-to-first-trimester NT-proBNP ratio of < 0.95 were 94.2%. As mentioned above, NT-proBNP can be a predictive marker of HDP development in early pregnancy. Taken together, these findings are helpful in HDP excluding and risk stratification in pregnant women, and it may facilitate follow-up in routine care as outpatients. In contrast, these findings have quite a low PPV. To address this issue, we propose the use of a combination of relatively less invasive methods for patients presenting with low NT-proBNP levels during early gestation and high third-to-first-trimester NT-proBNP ratios. For example, the strategies could involve monitoring home blood pressure and educating patients about the warning signs of HDP, such as headaches, blurred vision, and rapidly increasing swelling of the face, hands, or feet.

Additionally, our study showed that the HDP group had a significantly higher ratio of NT-proBNP levels from the third to the first trimester. A recent study reported that 546 healthy pregnant women in the United States had the highest NT-proBNP levels in the first trimester (median: 68 [range: 41–98] pg/mL), which decreased toward the second (median: 53 [range: 30–80] pg/mL) and third (median: 36 [range: 25–60] pg/mL) trimesters^26^. In addition, pregnant women in the first trimester had a higher NT-proBNP level than those in the third trimester^26^. Our study on women without HDP had similar results. However, data on women with HDP were comparable between the first and third trimester, which is a novel finding. Unlike the observed increase in NT-proBNP levels in non-HDP cases during the first trimester followed by a decline thereafter, no significant gradient was observed, at least in the first and third trimesters in HDP. Taken together, the hemodynamic profiles may differ between healthy pregnant women and those with HDP. Consequently, detailed monitoring of NT-proBNP levels could further elucidate the prognostic utility for HDP. Hence, the frequent measurement of NT-proBNP levels during pregnancy is necessary for future investigations.

The current study had several strengths. For example, it used a novel approach for evaluating the predictive value of early-pregnancy NT-proBNP levels in both nulliparous and multiparous women. This reinforces the findings of previous studies that investigated early-pregnancy NT-proBNP levels only in nulliparous women^21,47^. The abovementioned data emphasized the increased early-pregnancy NT-proBNP levels, suggesting the purpose of adjusting to pregnancy and indicating that NT-proBNP can be a possible predictor of HDP. We next plan to conduct a prospective study in our branch clinic to further investigate the observed associations between NT-proBNP and HDP.

The current study also had several limitations that should be acknowledged. This was a single-center retrospective cohort study with a small sample size and limited ethnicity (Japanese patients). These factors might have affected the study results. In addition, our clinic is located at a primary care hospital equipped to handle deliveries after 35 weeks of gestation. Hence, unlike a perinatal medical center, we could not manage cases before 35 weeks of gestation, especially those involving early-onset HDP, and severe HDP cases requiring intensive care, potentially leading to some discrepancies. It should be noted that our results apply exclusively to cases of HDP delivered after 35 weeks of gestation. In addition, our finding that the HDP group had a significantly higher ratio of NT-proBNP levels from the third to the first trimester is not applicable to cases of HDP developing before 35 weeks of gestation, as it necessitates NT-proBNP measurements in the third trimester of pregnant women diagnosed with HDP. Previous studies have revealed that maternal insufficiency of blood perfusion may be more critical in early-onset preeclampsia than in late-onset preeclampsia. Moreover, early-onset preeclampsia is more significantly affected by placental factors^4,48^; thus, the outcomes might be more impressive when focusing on early-onset preeclampsia. Therefore, a larger, multicenter study involving perinatal medical centers that may have managed several early-onset preeclampsia cases should be performed to validate our findings. Moreover, further research on the use of NT-proBNP levels and other examinations, such as assessment of predictive biomarkers and ultrasonography, must be conducted to enhance predictive power.

In conclusion, high early-pregnancy NT-proBNP levels were associated with a lower HDP risk. Further, a cutoff point of ≥ 60.5 pg/mL for early-pregnancy NT-proBNP levels had a high NPV and sensitivity for ruling out HDP, thereby emphasizing its potential as a predictive marker of HDP onset after 35 weeks of gestation. These findings can set the stage for a deeper exploration of the detailed mechanisms and clinical implications of the associations between NT-proBNP levels and HDP.

## Methods

### Study design and participants

A retrospective cohort study was conducted to evaluate the association between early- and late-pregnancy NT-proBNP levels and HDP diagnosed after 35 weeks of gestation. The medical records of the mothers and neonates were obtained from an electronic medical database. Informed consent was obtained to publish the information in an online open access publication. This study included pregnant women who delivered at our primary care obstetric clinic between April 2021 and March 2022. Patients were divided into two groups: those who developed HDP and those who did not. Based on the Japanese clinical guidelines for obstetrical practice^49^, prenatal visits are scheduled as follows: every 4 weeks after 12 weeks of gestation, biweekly between 24 and 35 weeks, and weekly after 36 weeks. We excluded patients with a history of cardiovascular diseases, renal disorders, or multiple pregnancies to eliminate potential confounding factors. Our clinic, which is a primary care hospital, refers patients with a gestational age of under 35 weeks who required specialized care at advanced perinatal medical centers. Therefore, we evaluated women who delivered after 35 weeks of gestation.

In this study, early pregnancy was defined as pregnancy in the first trimester, specifically from 7 to 13 weeks of gestation. Meanwhile, late pregnancy was defined as pregnancy in the third trimester, which is between 35 and 37 weeks of gestation.

### Diagnosis and management of HDP

Patients were diagnosed with HDP based on the 2018 International Society for the Study of Hypertension in Pregnancy (ISSHP) criteria^50^, and HDP included gestational hypertension and preeclampsia. There were no women with chronic hypertension or superimposed preeclampsia. Hypertension was defined as a systolic blood pressure of > 140 mmHg or diastolic blood pressure of > 90 mmHg on at least two occasions. Oral antihypertensive drugs, such as methyldopa, calcium blockers, and intravenous calcium blockers, were administered if necessary. Magnesium sulfate (MgSO4) was administered to patients with eclampsia or those at high risk of eclampsia to prevent seizures. Initially, 4 g of MgSO_4_ was intravenously administered for 30 min, followed by a continued dose of 1 g/h. Then, close monitoring of the serum magnesium level and related side effects was performed. The indication for delivery was the inability to control maternal blood pressure using antihypertensive drugs or 37 weeks of gestation. The attending physician selected the mode of delivery according to obstetric and fetal conditions.

### Blood collection and plasma NT-proBNP levels

Maternal blood samples were collected at two distinct stages: early pregnancy (7–13 weeks) and late pregnancy (35–37 weeks) using standard venipuncture methods. The patients provided nonfasting blood samples. At the Keihin Medical Laboratory (Kawasaki, Kanagawa, Japan), NT-proBNP levels were measured using the Roche Elecsys proBNP II electrochemiluminescence sandwich immunoassay with a Cobas e 801 analyzer (Roche Diagnostics, Rotkreuz, Switzerland), according to the manufacturer’s instructions. The NT-proBNP levels ranged from 5 to 35,000 pg/mL. If the NT-proBNP levels exceeded 125 pg/mL, which is the threshold for nonpregnant adults, the patients underwent additional cardiovascular evaluations, including echocardiography.

### Statistical analysis

Statistical analyses were performed using the Statistical Package for the Social Sciences software version 18.0 (SPSS Inc., Chicago, IL) and GraphPad Prism 8.2.1 (GraphPad software, San Diego, USA). Between-group differences were evaluated using the Student’s *t*-test or the Mann–Whitney U test for continuous variables and the chi-square test or the Fisher’s exact test for categorical variables. Spearman’s correlation coefficient was used to evaluate correlations. Receiver operating characteristic curve analysis was performed to assess the diagnostic performance of early-pregnancy NT-proBNP levels and its third-to-first-trimester ratio in predicting subsequent HDP. The logistic regression models were applied to evaluate the association between early-pregnancy NT-proBNP levels and HDP development using a reference group (patients without HDP). The models were adjusted for potential confounders such as age, BMI (in early pregnancy), aspirin use, parity, and systolic/diastolic blood pressure. Data were presented as crude and adjusted OR with 95% CIs. *p* values of < 0.05 were considered statistically significant. Other data were presented as mean ± standard error of the mean, mean ± standard deviation, median with range, or n (%).

### Study approval

The Ethical Committee of the Hagukumi Maternal and Child Clinic approved the current study. Informed consent was obtained from all participants prior to the study. We confirmed that all methods were performed in accordance with the relevant guidelines and regulations.

### Supplementary Information

Supplementary Table S1.

Supplementary Table S2.

## Data Availability

Data is provided within the manuscript or supplementary information files.
