# Author Correction: Early-pregnancy N-terminal pro-brain natriuretic peptide level is inversely associated with hypertensive disorders of pregnancy diagnosed after 35 weeks of gestation

**DOI:** 10.1038/s41598-024-68136-w

**Published:** 2024-08-06

**Authors:** Masaya Takahashi, Luka Suzuki, Nanase Takahashi, Mayu Hanaue, Masahiro Soda, Tamito Miki, Naoko Tateyama, Shiro Ishihara, Taro Koshiishi

**Affiliations:** 1 Hagukumi Maternal and Child Clinic, Kanagawa, Japan; 2 Department of Medicine, Diabetes Center, Quantitative Biosciences Institute (QBI), UCSF (University of California San Francisco), San Francisco, CA USA; 3 Department of Metabolism and Endocrinology, Juntendo University Graduate School of Medicine, Tokyo, Japan; 4 Department of Cardiology, Saitama Medical Center, Saitama Medical University, Saitama, Japan

Correction to: *Scientific Reports* 10.1038/s41598-024-63206-5, published online 28 May 2024

In the original version of this Article Figure 3c contained an incorrect marking. The original Figure 3 and accompanying legend appear below.

**Figure 3:**
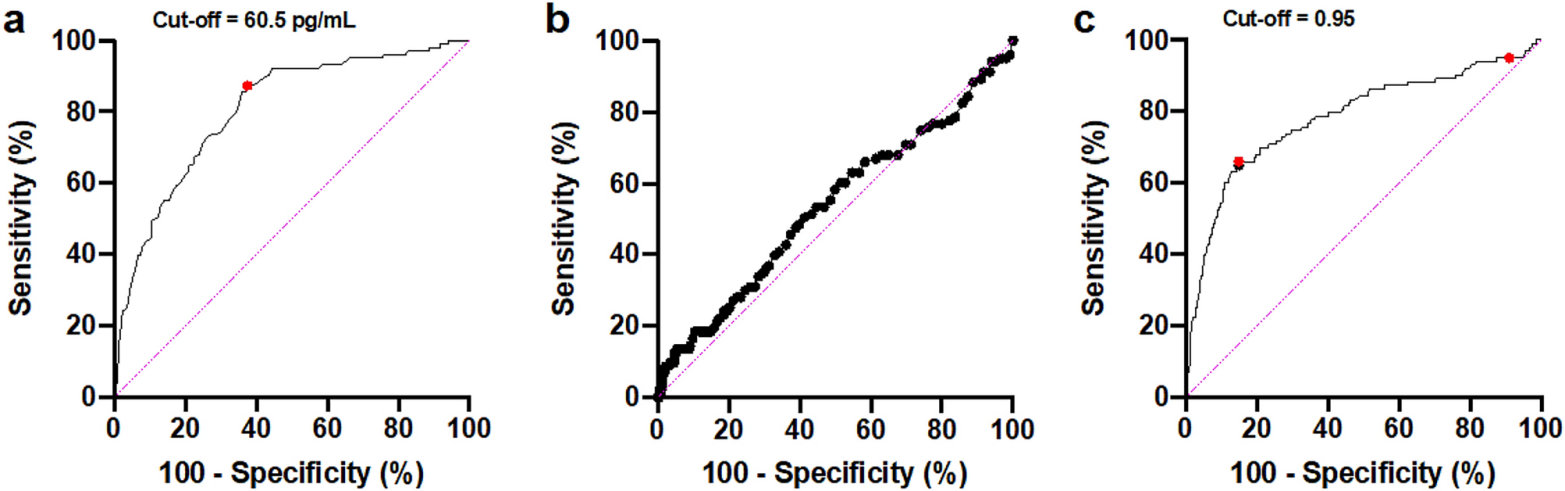
ROC curve for the accuracy analysis of NT-proBNP levels and the ratio of third-to-first-trimester NT-proBNP levels for predicting HDP. (**a**) NT-proBNP levels during the first trimester (7–13 weeks of gestation). (**b**) NT-proBNP levels during the third trimester (35–37 weeks of gestation). (**c**) Ratio of third-to-first-trimester NT-proBNP levels. ROC, receiver operating characteristic; NT-proBNP, N-terminal pro-brain natriuretic peptide; HDP, hypertensive disorders of pregnancy.

The original Article has been corrected.

