# Supplementary material for: Early-pregnancy N-terminal pro-brain natriuretic peptide level is inversely associated with hypertensive disorders of pregnancy diagnosed after 35 weeks of gestation
Source: Sci Rep. 2024 May 28;14:12225. doi: 10.1038/s41598-024-63206-5 (PMC11133404; doi:10.1038/s41598-024-63206-5)
Supplement: Supplementary file 1 — Supplementary Table S1. [file 41598_2024_63206_MOESM1_ESM.docx]

| **Supplementary Table 1. Crude and adjusted association of the first-trimester NT-proBNP and HDP** | | | | |
| --- | --- | --- | --- | --- |
| Measurement parameters | Crude OR (95% CI) | P value | Adjusted OR (95% CI) | P value |
| NT-proBNP levels (OR per 10 units) | 0.66 (0.60–0.73) | <0.0001 | 0.66 (0.59–0.72)^a^ | <0.0001 |

Abbreviations: NT-proBNP, N-terminal pro-brain natriuretic peptide; HDP, hypertensive disorder of pregnancy; BP, blood pressure; OR, odds ratio; CI, confidence interval.

Data were presented as OR with 95% CI.

^a^Adjusted for age, body mass index (in early pregnancy), parity, systolic/diastolic blood pressure, and aspirin use.

P-values <0.05 were considered statistically significant.
