# Supplementary material for: Early-pregnancy N-terminal pro-brain natriuretic peptide level is inversely associated with hypertensive disorders of pregnancy diagnosed after 35 weeks of gestation
Source: Sci Rep. 2024 May 28;14:12225. doi: 10.1038/s41598-024-63206-5 (PMC11133404; doi:10.1038/s41598-024-63206-5)
Supplement: Supplementary file 2 — Supplementary Table S2. [file 41598_2024_63206_MOESM2_ESM.docx]

| **Supplementary Table 2. Correlation analysis between NT-proBNP levels and basic indicators or outcomes** | | | | |
| --- | --- | --- | --- | --- |
|  | NT-proBNP levels in early pregnancy | | NT-proBNP levels in late pregnancy | |
|  | Spearman correlation | P value | Spearman correlation | P value |
| Maternal age | 0.007 | 0.853 | -0.006 | 0.860 |
| BMI |  |  |  |  |
| Early pregnancy | −0.180 | <0.0001 | −0.204 | <0.0001 |
| Late pregnancy | −0.141 | <0.0001 | −0.151 | <0.0001 |
| Blood pressure |  |  |  |  |
| Early pregnancy |  |  |  |  |
| Systolic | −0.156 | <0.0001 | −0.071 | <0.05 |
| Diastolic | −0.164 | <0.0001 | −0.027 | 0.451 |
| Late pregnancy |  |  |  |  |
| Systolic | −0.051 | 0.158 | −0.054 | 0.134 |
| Diastolic | −0.042 | 0.243 | −0.006 | 0.872 |
| Hb levels |  |  |  |  |
| Early pregnancy | −0.176 | <0.0001 | −0.082 | <0.05 |
| Late pregnancy | 0.069 | 0.050 | −0.133 | <0.001 |
| Gestational age at delivery | 0.018 | 0.613 | 0.007 | 0.836 |
| Blood loss volume | 0.005 | 0.895 | 0.053 | 0.140 |
| Birth weight | −0.054 | 0.136 | −0.041 | 0.262 |
| Umbilical artery pH | −0.044 | 0.223 | −0.015 | 0.673 |

Abbreviations: NT-proBNP, N-terminal pro-brain natriuretic peptide; BMI, body mass index.

Early pregnancy was defined as blood sampling during the first trimester (7–13 weeks of gestation).

Late pregnancy was defined as blood sampling during the third trimester (35–37 weeks of gestation).
